# Use of Mobile Information Technology during Planning, Implementation and Evaluation of a Polio Campaign in South Sudan

**DOI:** 10.1371/journal.pone.0135362

**Published:** 2015-08-07

**Authors:** John Haskew, Veronica Kenyi, Juma William, Rebecca Alum, Anu Puri, Yehia Mostafa, Robert Davis

**Affiliations:** 1 International Federation of Red Cross and Red Crescent Societies, Eastern African and Indian Ocean Islands Delegation, Nairobi, Kenya; 2 South Sudan Red Cross Society, Juba, South Sudan; 3 Ministry of Health, Juba, South Sudan; 4 UNICEF, Juba, South Sudan; 5 World Health Organization, Juba, South Sudan; 6 American Red Cross, Nairobi, Kenya; Kirby Institute, AUSTRALIA

## Abstract

**Background:**

Use of mobile information technology may aid collection of real-time, standardised data to inform and improve decision-making for polio programming and response. We utilised Android-based smartphones to collect data electronically from more than 8,000 households during a national round of polio immunisation in South Sudan. The results of the household surveys are presented here, together with discussion of the application of mobile information technology for polio campaign planning, implementation and evaluation in a real-time setting.

**Methods:**

Electronic questionnaires were programmed onto Android-based smartphones for mapping, supervision and survey activities during a national round of polio immunisation. National census data were used to determine the sampling frame for each activity and select the payam (district). Individual supervisors, in consultation with the local district health team, selected villages and households within each payam. Data visualisation tools were utilised for analysis and reporting.

**Results:**

Implementation of mobile information technology and local management was feasible during a national round of polio immunisation in South Sudan. Red Cross visits during the polio campaign were equitable according to household wealth index and households who received a Red Cross visit had significantly higher odds of being aware of the polio campaign than those who did not. Nearly 95% of children under five were reported to have received polio immunisation (according to maternal recall) during the immunisation round, which varied by state, county and payam. A total of 11 payams surveyed were identified with less than 90% reported immunisation coverage and the least poor households had significantly higher odds of being vaccinated than the most poor. More than 95% of households were aware of the immunisation round and households had significantly higher odds of being vaccinated if they had prior awareness of the campaign taking place.

**Conclusion:**

Pre-campaign community education and household awareness of polio is important to increase campaign participation and subsequent immunisation coverage in South Sudan. More emphasis should be placed on ensuring immunisation is equitable according to geographic area and household socio-economic index in future rounds. We demonstrate the utility of mobile information technology for household mapping, supervision and survey activities during a national round of polio immunisation and encourage future studies to compare the effectiveness of electronic data collection and its application in polio planning and programming.

## Introduction

Multiple types of surveys and methodologies are required to map polio immunisation coverage and collect data for planning, implementation and monitoring of polio immunisation campaigns. [1,2] These surveys often involve collection of a combination of geospatial and socio-demographic data from multiple households within multiple communities across different regions of a country. Sample sizes vary according to activity-specific methodologies, which include lot quality assurance sampling (LQAS) and cluster-based sampling, and hundreds of individual and household records are required to be completed on paper forms. [2,3] These paper forms must be printed, distributed to the field, completed, collected, and returned for data entry. Data must be manually double entered, by separate data entry clerks, into a computer database and compared to reconcile discordant entries against the original paper copy. The paper forms must then be stored securely for a minimum period of two or five years after publication according to national and international guidelines. [4,5] This process can be time consuming and costly to implement and there is potential to introduce error at each stage. There has been concern over the quality of polio supplementary immunisation activity (SIA) data, which may be delayed and incomplete due to errors in the above process, and can impede efforts to achieve polio eradication. Independent monitoring guidelines recommend that results of SIA monitoring be posted internationally within two weekly of each campaign. [2] The 2010–2012 Global Polio Eradication Initiative (GPEI) strategic plan emphasised that “*the gap in credible and timely SIA coverage data to assess risks and guide improvements has been a continuing constraint in both endemic and in re-infected countries*.*”* [6]

Adoption of standardised guidelines and methodologies, and access to timely and reliable data, thus remains a challenge for polio immunisation programs, particularly in insecure and inaccessible areas. Use of mobile information technology may help in enabling standardised data to be collected in real-time, and several advantages have been discussed that could help to inform and improve decision-making for polio programming and response. Electronic data collection has been proposed as a solution to many challenges faced by paper-based surveys and is increasingly being adopted in a number of public health programs, including the integrated management of childhood illness [7], community epidemiology [8] and neglected tropical diseases. [9] Moreover, open source applications hosted on the Android (Google Inc.) platform, including Open Data Kit (ODK; www.opendatakit.org) [10], have enabled public health managers and epidemiologists with limited software programming experience to utilise mobile information technology for public health programming. Android-based smartphones offer additional capabilities including built-in global positioning systems (GPS) functionality and other applications that can be integrated into electronic data collection, including barcode scanning, digital photography and automated timestamp information. Mobile information technology also enables other data sources, including census and mapping data, and other tools for data visualisation, including Google Maps [11], to be more readily integrated into the process of data collection, reporting and analysis. [12,13]

## Methods

### Study setting

South Sudan is situated in the “wild poliovirus importation belt”, a band of countries stretching from West Africa to the Horn of Africa, which are vulnerable to re-infection with imported poliovirus. In May 2013, the Horn of Africa, which had been polio-free for several years, suffered an importation of wild polio virus that first affected Somalia and then rapidly spread to the neighbouring countries of Kenya and Ethiopia. [14] South Sudan is at high risk of an outbreak given its borders with Kenya and Ethiopia, low rates of vaccination coverage and population displacement following recent insecurity and internal conflict.

An emergency national round of polio immunisation took place in South Sudan in November 2013, led by the Ministry of Health. The South Sudan Red Cross Society supported this campaign by mobilising 840 volunteers to conduct community and household-based education and awareness in 24 counties of Western, Central and Eastern Equatoria states. The International Federation of Red Cross and Red Crescent Societies (IFRC) provided technical support to SSRC and supported national training of trainers of 52 Red Cross supervisors in Juba. Supervisors were trained in the application of mobile information technology for mapping, survey methodology and electronic data collection. Each Red Cross supervisor was responsible for 20 volunteers (working in teams of two) and was allocated payams (districts) in which to work during the polio campaign. A one day training of Red Cross volunteers took place in the respective payams prior to activities starting in November 2013. Due to on-going insecurity in South Sudan, this report only includes results from 41 supervisors (and their smartphones), which were returned in time for the publication of this study.

### Study design

For four days prior to the November 2013 polio immunisation campaign, Red Cross volunteers conducted community and household visits to raise awareness of the campaign and importance of children being vaccinated. An estimated 252,078 households and 428,533 children under five were reached during social mobilisation activities. [15] At the same time, Red Cross supervisors (each responsible for 20 volunteers who worked in teams of two) conducted pre-campaign household surveys using an Android-based smartphone to map immunisation coverage and identify areas not included in prior vaccination campaigns.

Intra-campaign monitoring took place during the four days of the polio immunisation campaign itself. Supervisors were allocated one or two payams during the national training and each was responsible, in consultation with the local district health team, for selecting a minimum of four villages within each payam for monitoring. Each supervisor then used simple random sampling to select ten households in each village for interview. South Sudan national census data from 2010 [16] were used to select and allocate payams to individual supervisors and payam level information was included within the electronic questionnaires to enable geocoding of households to take place independently of collection of GPS co-ordinates.

The post-campaign survey took place over two days immediately following the polio immunisation campaign and a two-stage cluster random sampling design was used to select the sample. In the first stage, 200 clusters (payams) were randomly selected with probability proportional to the estimated population using computer generated random numbers. National census data were used to determine the sampling frame. [16] In the second stage, simple random sampling was used by the supervisor to select twenty households from within each cluster to be interviewed.

### Data collection and management

Electronic questionnaires were designed in extended mark-up language (XML) and uploaded to Android-based smartphones for activities of pre-campaign mapping, intra-campaign supervision and post-campaign survey. Each questionnaire was programmed for the Open Data Kit (ODK) application and skip logic algorithms were used. GPS co-ordinates of each household were recorded as part of the questionnaire. All household interviewers received appropriate training in the use of mobile phones and appropriate interview techniques. Data verification was used to ensure information entered was valid and the system provided the flexibility to have multiple language translations.

Due to the emergency nature of the polio campaign in South Sudan, it was not possible to establish a data contract with the mobile phone operator prior to the start of the campaign and so data were not uploaded in real-time. Instead, data were uploaded from the Android-based smartphones to a server on their return to Nairobi and this database was subsequently downloaded, coded and cleaned. Data were imported into STATA version 12.1 (Stata Corp; College Station, TX) and restructured using the reshape command according to the number of children under five residing in each household.

### Statistical analysis

A relative index of socio-economic status for each household was calculated based on 16 variables that coded household construction (floors, walls, roof) and assets. [17,18] Principal component analysis was used to define an appropriate weight for each item. [19] The index is the first principal component, since it summarises the largest amount of information common to household construction and assets. The first principal component explained 20.8% of the variability in the 16 variables and gave greatest weight to the household floor constructed of cement (0.44), roof constructed of iron sheets (0.36), walls constructed of bricks or blocks (0.26) and ownership of a phone (0.23) in the household. The first three eigenvalues were 3.32, 1.67, and 1.47 and accounted for 20.8%, 10.5%, and 9.2% of the variation, respectively.

Associations between variables were considered significant at p-value < 0.05 in univariable analysis. Odds Ratios, with associated 95% confidence intervals and p-values, are presented where appropriate. All data were analysed using STATA version 12.1.

### Ethics statement

This was an emergency public health program designed to increase polio vaccination coverage in South Sudan, in line with goals set by the Ministry of Health and Government of South Sudan, and thus did not require institutional review board (IRB) and ethical approvals. Informed written consent for participation in each survey was obtained from each household by the Red Cross volunteer. All data were anonymised prior to analysis. The study was approved by the Director of Public Health, Ministry of Health, South Sudan.

## Results

### Pre-campaign mapping

A total of 4,415 children under five, 2,850 households and 56 payams were included in the pre-campaign mapping exercise. All households were geocoded to the level of the payam and just under half of households surveyed had GPS co-ordinates recorded. Reported polio immunisation coverage (by maternal recall) was above 80% (prior to the November 2013 campaign), which varied by state, county and payam (Table 1). A total of 25 payams mapped were identified with less than 90% reported immunisation coverage (by maternal recall) during previous rounds (Table 1). Households had more than seven times higher odds of being vaccinated if they had prior awareness of the campaign (OR 6.8, 95% CI 5.6–8.3, p < 0.001) and the least poor households were more likely be vaccinated than the most poor (OR 2.5, 95% CI 1.9–3.2, p < 0.001) (Table 2). The least poor households were also more likely to be aware of the polio campaign than the most poor (OR 5.6, 95% CI 4.1–7.6, p = 0.001) (Table 3).

**Table 1:** Reported polio immunisation coverage (n / N) according to state, county and payam (pre- and post- November 2013 polio campaign).

			Pre- November 2013 campaign		Post- November 2013 campaign	
State	County	Payam	% immunised	n / N	% immunised	n / N
Central Equatoria	Juba	Bungu	100.00%	53 / 53		
		Dolo			100.00%	120 / 120
		Ganji	88.90%	24 / 27		
		Northern Bari	100.00%	20 / 20	100.00%	112 / 112
		Rejaf	84.20%	16 / 19		
		Rokon	100.00%	22 / 22		
		Sakure			63.50%	61 / 96
	Kajo Keji	Kangapo I			100.00%	44 / 44
	Lainya	Wonduruba	100.00%	30 / 30	100.00%	95 / 95
		Kenyi	90.40%	75 / 83	100.00%	51 / 51
		Lainya	71.60%	73 / 102	94.30%	83 / 88
		Mukaya	93.90%	46 / 49	100.00%	20 / 20
	Terekeka	Wuji	79.00%	15 / 19	100.00%	21 / 21
		Muni	96.20%	75 / 78	97.30%	73 / 75
		Nyori	100.00%	20 / 20	97.80%	45 / 46
		Reggo	95.00%	57 / 60	79.70%	47 / 59
		Rijong	100.00%	27 / 27		
		Tombek			100.00%	21 / 21
	Yei	Terekeka	91.30%	21 / 23		
		Lasu	84.80%	50 / 59	100.00%	27 / 27
		Otogo	97.20%	105 / 108	100.00%	114 / 114
		Tore	98.00%	50 / 51	96.30%	26 / 27
Eastern Equatoria	Budi	Kimotong	71.00%	22 / 31		
		Komori	90.00%	45 / 50	81.80%	45 / 55
		Nagishot			98.30%	115 / 117
		Napak	96.60%	226 / 234		
	Ikotos	Lomohidang South	87.50%	14 / 16	93.10%	54 / 58
		Losite	84.20%	16 / 19	100.00%	20 / 20
	Kapoeta East	Katodori	0.00%	0 / 110	92.70%	101 / 109
		Narus	96.70%	58 / 60	100.00%	61 / 61
	Kapoeta North	Chumakori	89.60%	146 / 163	76.80%	53 / 69
		Lomeyen	96.40%	54 / 56	100.00%	63 / 63
		Najie	92.10%	35 / 38	70.70%	29 / 41
		Paringa	83.00%	78 / 94	90.80%	69 / 76
	Lopa	Burgilo	100.00%	34 / 34		
	Torit	Hiyala	96.60%	85 / 88	100.00%	18 / 18
		Ifwotu	94.70%	18 / 19	98.60%	71 / 72
Western Equatoria	Ibba	Ibba Centre	60.60%	40 / 66		
		Madebe	53.60%	30 / 56		
		Nabanga	100.00%	33 / 33	97.30%	36 / 37
	Maridi	Kozi	100.00%	37 / 37	100.00%	32 / 32
		Landili	86.20%	25 / 29		
		Maridi	66.00%	103 / 156	95.70%	179 / 187
		Ngamunde	38.90%	7 / 18	96.00%	24 / 25
	Mundri East	Kedi 'ba	72.70%	136 / 187	100.00%	109 / 109
		Lakamadi	100.00%	26 / 26		
	Mundri West	Amadi	100.00%	39 / 39	84.20%	16 / 19
		Kotobi	100.00%	32 / 32	92.30%	36 / 39
		Mundri	100.00%	184 / 184	100.00%	87 / 87
	Mvolo	Lessi			100.00%	169 / 169
		Kokor	96.80%	90 / 93		
		Mvolo	100.00%	47 / 47	100.00%	98 / 98
		Yeri			98.00%	49 / 50
	Nzara	Basukangbi	100.00%	61 / 61	100.00%	12-Dec
		Ringasi	87.50%	49 / 56	83.90%	26 / 31
		Sakure	55.20%	48 / 87		
		Sangua	99.40%	162 / 163	100.00%	24 / 24
	Tambura	Mupoi	78.90%	41 / 52	90.90%	60 / 66
		Source Yubu	86.80%	46 / 53	98.50%	67 / 68
		Tambura	74.10%	83 / 112	82.10%	69 / 84
	Yambio	Nadiangere	98.70%	76 / 77	77.10%	37 / 48
		Ri-Rangu	81.00%	34 / 42	78.60%	22 / 28
		Yambio	83.60%	178 / 213	81.70%	210 / 257

**Table 2:** Univariable associations of reported polio immunisation coverage with household awareness of the campaign, socio-economic index and child age (pre- and post- November 2013 polio campaign).

	Pre- November 2013 campaign				Post- November 2013 campaign			
	% of children under five immunised	n / N	OR (95% CI)	P	% of children under five immunised	n / N	OR (95% CI)	P
Total	83.80%	3,699 / 4,415			94.2%	3,764 / 3,998		
Household awareness of the campaign	** **	** **	** **	** **	** **	** **	** **	** **
No	51.60%	260 / 504	1		74.70%	127 / 170	1	
Yes	87.90%	3,439 / 3,911	6.84 (5.60–8.35)	<0.001	95.00%	3,637 / 3,828	6.45 (4.43–9.38)	<0.001
Wealth Index	** **	** **	** **	** **	** **	** **	** **	** **
Most poor	79.40%	911 / 1,148	1		93.10%	826 / 887	1	
Very poor	85.90%	567 / 660	1.59 (1.22–2.07)	0.001	93.60%	612 / 654	1.08 (0.72–1.62)	0.72
Poor	80.00%	807 / 1,009	1.04 (0.84–1.28)	0.72	90.20%	721 / 799	0.68 (0.48–0.97)	0.03
Less Poor	85.70%	571 / 666	1.56 (1.21–2.03)	0.001	96.90%	719 / 742	2.31 (1.41–3.77)	0.001
Least Poor	90.50%	843 / 932	2.46 (1.90–3.20)	<0.001	96.70%	886 / 916	2.18 (1.39–3.41)	0.001
Child age	** **	** **	** **	** **	** **	** **	** **	** **
< 6 months	74.10%	547 / 738	1		86.10%	482 / 560	1	
6–12 months	84.60%	717 / 848	2.07 (1.65–2.60)	<0.001	96.10%	1,023 / 1,065	3.94 (2.67–5.82)	<0.001
12–24 months	85.60%	1,103 / 1,289	2.24 (1.79–2.79)	<0.001	96.40%	1,638 / 1,700	4.27 (3.02–6.06)	<0.001
24–59 months	86.50%	1,332 / 1,540	1.91 (1.49–2.45)	<0.001	92.30%	621 / 673	1.93 (1.33–2.80)	<0.001

**Table 3:** Univariable associations of household awareness of polio campaigns with socio-economic index and Red Cross household visit.

	% Household awareness	n / N	OR (95% CI)	P
Wealth Index				
Most poor	74.0%	850 / 1,148	1	
Very poor	96.2%	635 / 660	8.90 (5.85–13.56)	<0.001
Poor	93.3%	941 / 1,009	4.85 (3.67–6.41)	<0.001
Less Poor	91.3%	608 / 666	3.68 (2.72–4.96)	<0.001
Least Poor	94.1%	877 / 932	5.59 (4.13–7.57)	<0.001
Red Cross visit				
No	80.0%	112 / 140	1	
Yes	98.5%	2,838 / 2,880	16.89 (10.10–28.24)	<0.001

### Intra-campaign supervision

**Fig 1:**
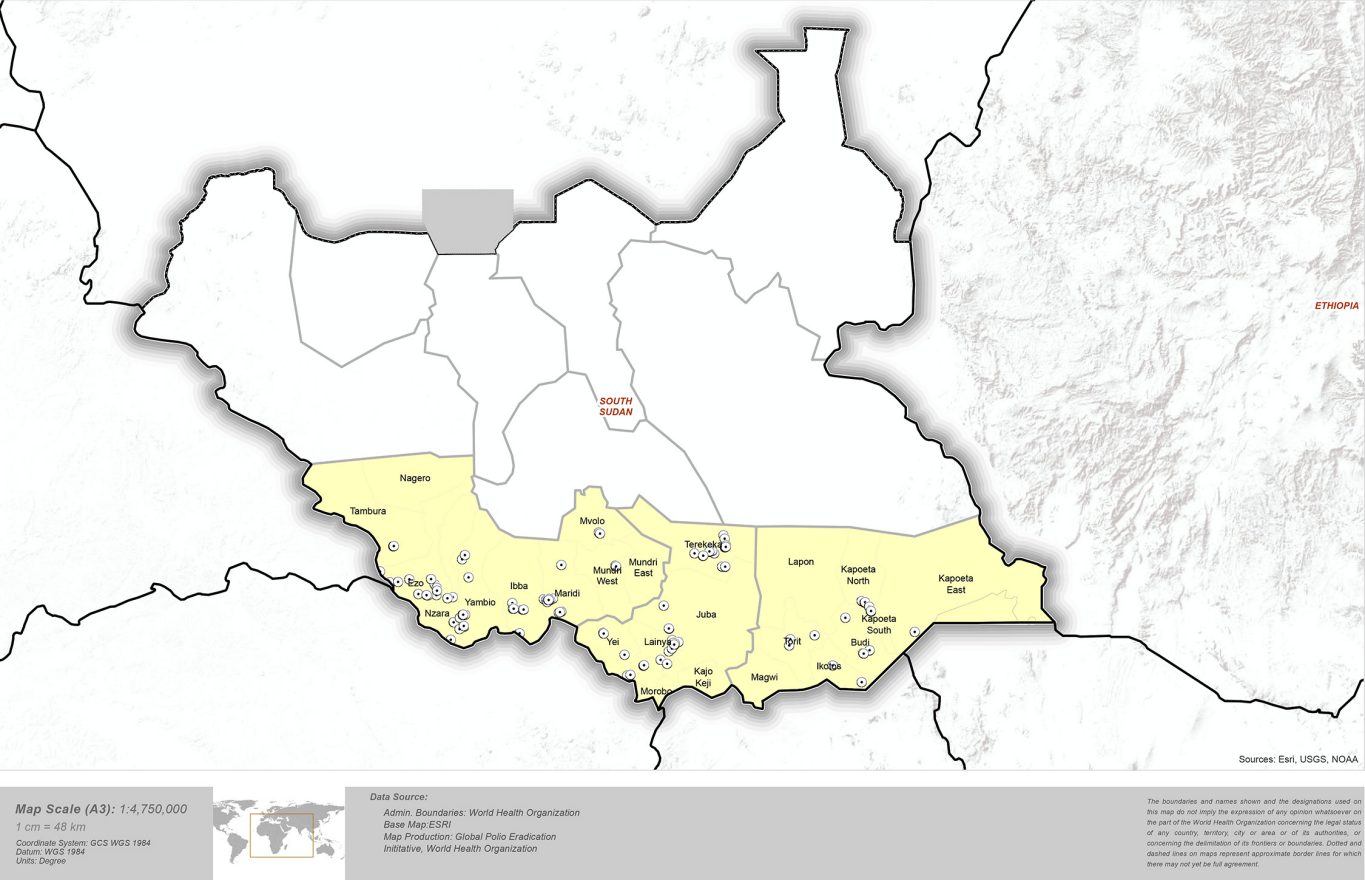
Map of South Sudan with dots representing households mapped during supervision activities*. * 49.0% of households had GPS co-ordinates recorded.

During community and household visits, Red Cross supervisors conducted household surveys to monitor Red Cross volunteer activities during social mobilisation and education activities. A total of 3,020 households and 60 payams were included in the intra-campaign supervision exercise. All households were geocoded to the level of the payam and nearly half of households had GPS co-ordinates recorded (Fig 1). Nearly 98% of households reported receiving a Red Cross visit during supervision monitoring. Households that received a Red Cross visit had significantly higher odds of being aware of the polio campaign than those who did not (OR 16.9, 95% CI 10.1–28.2, p < 0.001) (Table 3).

### Post-campaign survey

**Fig 2:**
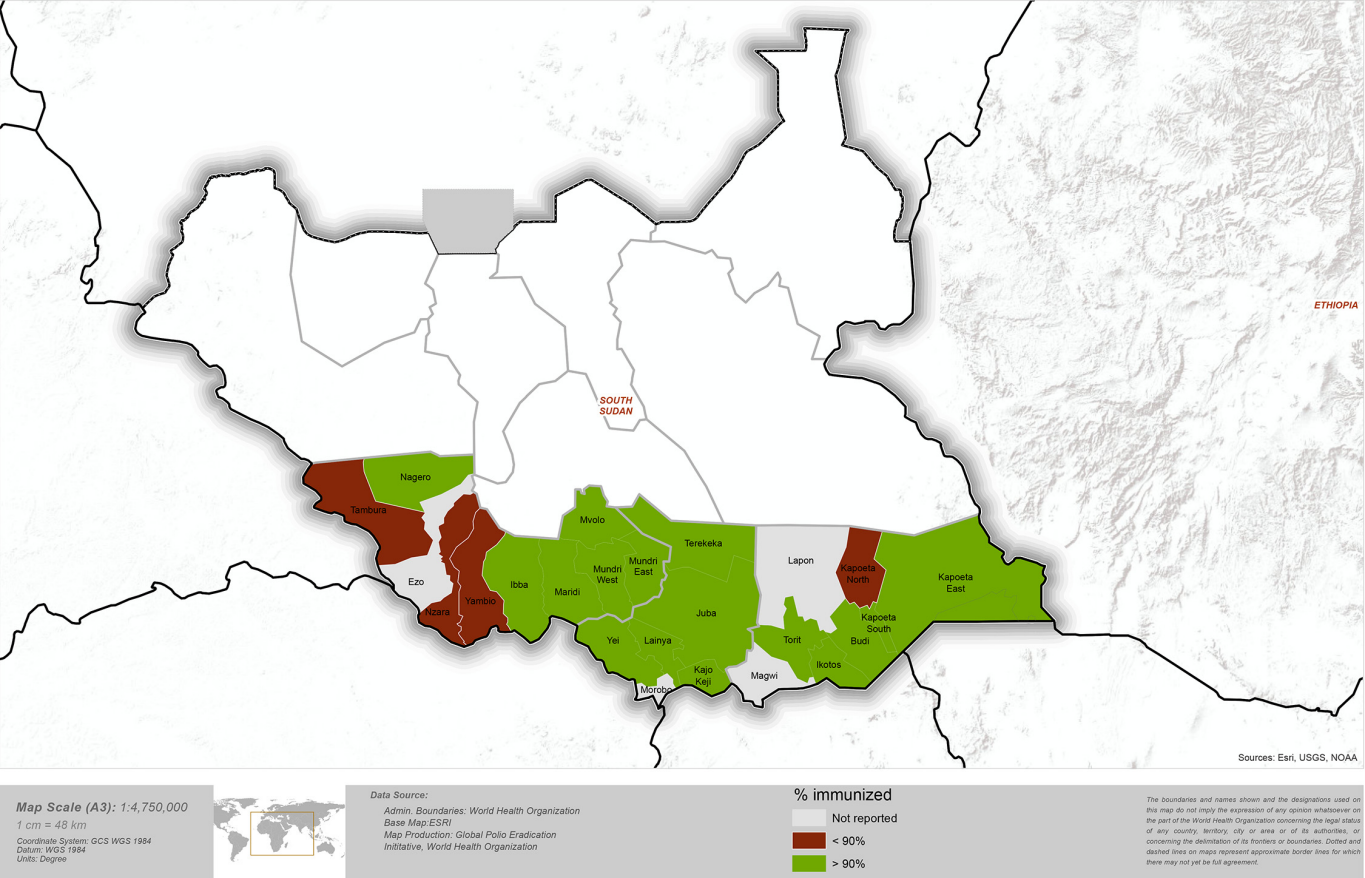
Map of South Sudan with colour representing reported post-campaign polio immunisation coverage. Red Colour < 90% reported polio immunisation coverage, Green Colour > 90% reported polio immunisation coverage.

For four days after the November 2013 polio campaign, Red Cross supervisors conducted a post-campaign survey to determine education and immunisation coverage. A total of 3,998 children under five, 2,540 households and 48 payams were surveyed during this exercise. All households were geocoded to the level of the payam and more than half of households had GPS co-ordinates recorded. Nearly 95% of children under five were reported to have received polio immunisation during the November immunisation round, which varied by state, county and payam (Table 1, Fig 2). A total of 11 payams surveyed were identified with less than 90% reported immunisation coverage of children under five (Table 1). The least poor households had significantly higher odds of being vaccinated than the most poor (OR 2.2, 95% CI 1.4–3.4, p = 0.001) (Table 2). 95.6% of households were aware of the November 2013 immunisation round and households had significantly higher odds of being vaccinated if they had prior awareness of the campaign taking place (OR 6.4, 95% CI 4.4–9.4, p < 0.001) (Table 3).

## Discussion

In the context of the South Sudan polio program, this study has highlighted the importance of pre-campaign community education and awareness of polio in increasing campaign participation and subsequent immunisation coverage. Eleven payams were reported to have less than 90% immunisation coverage following the November 2013 polio campaign, which should be considered during planning of future immunisation rounds. More emphasis should be placed on ensuring immunisation is equitable according to geographic area and household socio-economic index in future rounds. Pre-campaign mapping of areas missed during previous rounds of immunisation and post-campaign monitoring of education and immunisation coverage, particularly among high-risk areas and population groups, is encouraged.

It is important to acknowledge the methodological limitations of this study. First, the analyses presented do not account for the cluster sampling design and only univariable associations are presented. Reported results are also subject to the potential of both reporter and respondent bias, that may over-estimate the coverage of Red Cross education visits and polio campaign immunisation coverage. Selection of villages and households within each payam was made by individual supervisors, in consultation with the local district health team, and may not be representative of the payam as a whole and the sample size within some payams may not be powered to provide statistically significant results for that payam. Differential access to payams (for example, poor road infrastructure or rural location) may also have affected results of both immunisation and survey teams.

The use of mobile information technology during the polio campaign enabled standardised, coded data to be collected and analysed, and has the potential to inform polio planning and programming at different levels of decision-making. Electronic data collection has the potential to more readily integrate other tools including GPS, barcode, photographic and timestamp information and to save time and cost compared to paper-based alternatives. The technology could be readily applied to other polio program activities, for example acute flaccid paralysis (AFP) case surveillance, logistics and cold chain monitoring. Future adoption of this technology in other settings would need to take into account the mobile data network infrastructure as well as local guidelines on data management and security. Additional training and education needs of end users must also be considered. Less than half of all households surveyed had GPS co-ordinates recorded, which may highlight a gap in training and supervision. Further studies to compare the effectiveness of electronic data collection and other applications in polio planning and programming are encouraged.

## Supporting Information

S1 Consent Form Participant Consent Form for Household-based Survey. (DOC)

S1 Survey Questionnaire Red Cross Polio Mapping, Supervision and Post-campaign Survey Questionnaires. (DOC)
