# Supplementary material for: Use of Mobile Information Technology during Planning, Implementation and Evaluation of a Polio Campaign in South Sudan
Source: PLoS One. 2015 Aug 7;10(8):e0135362. doi: 10.1371/journal.pone.0135362 (PMC4529202; doi:10.1371/journal.pone.0135362)
Supplement: S1 Consent Form — (DOC) [file pone.0135362.s001.doc]

**PARTICIPANT CONSENT FORM**

Version 2.0. 25 October 2013

**Evaluation of a polio campaign in South Sudan**

**Household-based Survey**

**Lead Researcher: Dr John Haskew**

Please initial all boxes

1. I confirm that I have had the opportunity to consider the information provided by the surveyor, ask questions and have had these answered satisfactorily.

1. I understand that my participation is voluntary and that I am free to withdraw at any time without giving any reason, without my participation in the polio campaign being affected.
2. I agree to take part in the above study.

Name of Participant Date Signature

Name of Person Date Signature

obtaining consent
