# Supplementary material for: Use of Mobile Information Technology during Planning, Implementation and Evaluation of a Polio Campaign in South Sudan
Source: PLoS One. 2015 Aug 7;10(8):e0135362. doi: 10.1371/journal.pone.0135362 (PMC4529202; doi:10.1371/journal.pone.0135362)
Supplement: S1 Survey Questionnaire — (DOC) [file pone.0135362.s002.doc]

**Supplementary Material 1. Red Cross Polio Mapping Questionnaire**

| **INFO: Please confirm this population group is NOT included in the Red Cross micro-planning data** |
| --- |

| **No** | **Question** | **Responses** | | | **Response Type** |
| --- | --- | --- | --- | --- | --- |
| Q1 | Select Zone | [zones] | | | Single Select |
| Q2 | Select Woreda / County | [woredas / counties] | | | Single Select |
| Q3 | Collect GPS position | [gps] | | | [gps] |
|  |  |  | | |  |
|  | | | | | |
| **INFO: Please obtain the following information from the village chief / head** | | | | | |
|  | | | | | |
| Q4 | ASK: What is the name of the settlement? | |  | | String |
| Q5 | ASK: Did the population group receive Polio vaccination during the last round? | | Yes / No | | Boolean |
| Q6 | ASK: What type of population group live here? | | Permanent (e.g. village) | | Single Select |
|  |  | | Semi-permanent (e.g. seasonal settlement) | |  |
|  |  | | Temporary (e.g. refugee, displaced) | |  |
| Q7 | ASK: What type of temporary population groups are present? | | Refugee | | Single Select |
|  |  |  | Internally Displaced Person | |  |
|  | ***[If response to Q6 is Temporary]*** | | Nomadic | |  |
| Q8 | ASK: How long has the population been living in the current location? | | Less than 1 month | | Single Select |
|  |  |  | 1 month to 6 months | |  |
|  |  | | 6 months to 1 year | |  |
|  |  | | More than one year | |  |
| Q9 | ASK: Is there a history of travel of this population group in the last month? | | Yes / No | | Boolean |
| Q10 | ASK: Where has the population travelled in the past month?  ***[If response to Q9 is Yes]*** | | Within the Woreda / County | | Single Select |
|  |  |  | Within the Zone | |  |
|  |  | | Within the Region  Across an international border | |  |
| Q11 | ASK: What are the ethnicities of people who live here? | | [ethnicities] | | Multiple Select |
| Q12 | ASK: How many households are present here? | | | | Integer |
| Q13 | ASK: What is the estimated population that live here? | | | | Integer |
|  |  |  | | |  |
| **INFO: We will now survey 10 households. Start from the centre of the population group and spin a bottle to determine your direction.** | | | | | |
| **INFO: Start with the first household closest to the centre of the population group.** | | | | | |
|  |  |  | |  | |
| **[For each household]** | | | | | |
| Q14 | CHECK: Does a child under five live in household [x]? | Yes / No | | | Boolean |
| Q15 | ASK: How many children under five live in household [x]? | | | | Integer |
| **[For each child in the household]** | | | | | |
| Q16 | ASK: What is the age group of child [x]? | Less than 6 months | | | Single Select |
|  |  | 6 months to 1 year | | |  |
|  |  | 1 to 2 years | | |  |
|  |  | 2 to 5 years | | |  |
| Q17 | ASK: Was child [x] vaccinated for Polio during the last round? | Yes / No | | | Boolean |
| Q18 | ASK: Why was child [x] not vaccinated during the last round? | Child / Parents Not Available | | | Single Select |
|  | ***[If response to Q17 is No]*** | Team did not Visit the house | | |  |
|  |  | Child available in house but not vaccinated | | |  |
|  |  | Child Asleep | | |  |
|  |  | New Born | | |  |
|  |  | Visitor | | |  |
|  |  | Child sick | | |  |
|  |  | Other | | |  |
|  |  | Refused | | |  |
| Q19 | ASK: Why was child [x] refused to be vaccinated for Polio during the last round? | Religious | | | Single Select |
|  |  | No faith in vaccine | | |  |
|  | ***[If response to Q18 is Refused]*** | Decision maker not at home | | |  |
|  |  | Fatigue | | |  |
|  |  | Rumour | | |  |
|  |  | Demand for other services | | |  |
|  |  | No response / Other | | |  |
| **[End of each child in the household]** | | | | | |
|  | | | | | |
| Q20 | OBSERVE: Was the house marked by vaccination team? | Yes / No | | | Boolean |
| Q21 | OBSERVE: What material is the floor of the house made of? | Mud | | | Single Select |
|  |  | Cement | | |  |
|  |  | Other | | |  |
| Q22 | OBSERVE: What material are the walls of the house made of? | Earth | | | Single Select |
|  |  | Stone | | |  |
|  |  | Iron Sheets | | |  |
|  |  | Cement | | |  |
|  |  | Timber/Wood | | |  |
|  |  | Bricks/Blocks | | |  |
|  |  | Cardboard/Cartons | | |  |
| Q23 | OBSERVE: What material is the roof of the house made of? | Iron Sheets | | | Single Select |
|  |  | Cement | | |  |
|  |  | Grass/Makuti | | |  |
|  |  | Tiles | | |  |
|  |  | Timber/Wood | | |  |
|  |  | Other | | |  |
| Q24 | ASK: Was the household aware of the last polio campaign before start of it? | Yes / No | | | Boolean |
| Q25 | ASK: What was the household source of information about the last polio campaign? | Radio | | | Single Select |
|  |  | TV | | |  |
|  | ***[If response to Q24 is Yes]*** | MoH Health Staff / Vaccinators | | |  |
|  |  | Women / Youth group activities | | |  |
|  |  | Red Cross volunteers | | |  |
|  |  | Other Community mobilisers | | |  |
|  |  | Megaphones | | |  |
|  |  | Mosque Announcements / Religious leaders | | |  |
|  |  | SMS / Mobile | | |  |
|  |  | Other | | |  |
| Q26 | ASK: What assets do the household have: | Electricity | | | Multiple Select |
|  |  | Radio | | |  |
|  |  | Television | | |  |
|  |  | Phone | | |  |
|  |  | Fridge | | |  |
|  |  | Solar Panel | | |  |
|  |  | None | | |  |
|  |  | No response | | |  |
| Q27 | ASK: What is the highest level of school the mother has completed? | No formal education | | | Single Select |
|  |  | Islamic | | |  |
|  |  | Primary | | |  |
|  |  | Secondary | | |  |
|  |  | Higher | | |  |
|  |  | Mother not present | | |  |
| Q28 | ASK: What is the occupation of the mother? | Not employed | | | Single Select |
|  |  | Housewife | | |  |
|  |  | Self-employed | | |  |
|  |  | Employed by other business | | |  |
|  |  | Professional e.g. Teacher/Lawyer | | |  |
|  |  | Casual Labourer | | |  |
|  |  | Farmer | | |  |
|  |  | Other | | |  |
|  |  | Mother not present | | |  |
| **[End of each household]** | | | | | |

**Supplementary Material 2. Red Cross Polio Supervision Questionnaire**

| **No** | **Question** | **Responses** | | | **Response Type** |
| --- | --- | --- | --- | --- | --- |
| Q1 | Select Zone | [zones] | | | Single Select |
| Q2 | Select Woreda / County | [woredas / counties] | | | Single Select |
| Q3 | Collect GPS position | [gps] | | | [gps] |
|  |  |  | | |  |
|  | | | | | |
| **INFO: Please obtain the following information from the village chief / head** | | | | | |
|  | | | | | |
| Q4 | ASK: What is the name of the settlement? | |  | | String |
| Q5 | ASK: Was this village visited by a Red Cross volunteer? | | Yes / No | | Boolean |
| Q5 | ASK: Did the population group receive Polio vaccination during the last round? | | Yes / No | | Boolean |
| Q5 | ASK: When did the village receive a RC volunteer visit? | |  | |  |
| Q6 | ASK: What type of population group live here? | | Permanent (e.g. village) | | Single Select |
|  |  | | Semi-permanent (e.g. seasonal settlement) | |  |
|  |  | | Temporary (e.g. refugee, displaced) | |  |
| Q7 | ASK: What type of temporary population groups are present? | | Refugee | | Single Select |
|  |  |  | Internally Displaced Person | |  |
|  | ***[If response to Q6 is Temporary]*** | | Nomadic | |  |
| Q8 | ASK: How long has the population been living in the current location? | | Less than 1 month | | Single Select |
|  |  |  | 1 month to 6 months | |  |
|  |  | | 6 months to 1 year | |  |
|  |  | | More than one year | |  |
| Q9 | ASK: Is there a history of travel of this population group in the last month? | | Yes / No | | Boolean |
| Q10 | ASK: Where has the population travelled in the past month?  ***[If response to Q9 is Yes]*** | | Within the Woreda / County | | Single Select |
|  |  |  | Within the Zone | |  |
|  |  | | Within the Region  Across an international border | |  |
| Q11 | ASK: What are the ethnicities of people who live here? | | [ethnicities] | | Multiple Select |
| Q12 | ASK: How many households are present here? | | | | Integer |
| Q13 | ASK: What is the estimated population that live here? | | | | Integer |
|  |  |  | | |  |
| **INFO: We will now survey 10 households. Start from the centre of the population group and spin a bottle to determine your direction.** | | | | | |
| **INFO: Start with the first household closest to the centre of the population group.** | | | | | |
|  |  |  | |  | |
| **[For each household]** | | | | | |
| Q14 | OBSERVE: What material is the floor of the house made of? | Mud | | | Single Select |
|  |  | Cement | | |  |
|  |  | Other | | |  |
| Q15 | OBSERVE: What material are the walls of the house made of? | Earth | | | Single Select |
|  |  | Stone | | |  |
|  |  | Iron Sheets | | |  |
|  |  | Cement | | |  |
|  |  | Timber/Wood | | |  |
|  |  | Bricks/Blocks | | |  |
|  |  | Cardboard/Cartons | | |  |
| Q16 | OBSERVE: What material is the roof of the house made of? | Iron Sheets | | | Single Select |
|  |  | Cement | | |  |
|  |  | Grass/Makuti | | |  |
|  |  | Tiles | | |  |
|  |  | Timber/Wood | | |  |
|  |  | Other | | |  |
| Q17 | ASK: Was the household visited by a Red Cross volunteer in the past week? | Yes / No | | | Boolean |
| Q18 | ASK: What was the household source of information about the last polio campaign? | Radio | | | Single Select |
|  |  | TV | | |  |
|  |  | MoH Health Staff / Vaccinators | | |  |
|  |  | Women / Youth group activities | | |  |
|  |  | Red Cross volunteers | | |  |
|  |  | Other Community mobilisers | | |  |
|  |  | Megaphones | | |  |
|  |  | Mosque Announcements / Religious leaders | | |  |
|  |  | SMS / Mobile | | |  |
|  |  | Other | | |  |
| Q19 | ASK: What assets do the household have: | Electricity | | | Multiple Select |
|  |  | Radio | | |  |
|  |  | Television | | |  |
|  |  | Phone | | |  |
|  |  | Fridge | | |  |
|  |  | Solar Panel | | |  |
|  |  | None | No response | | |  |
| Q20 | ASK: What is the highest level of school the mother has completed? | No formal education | | | Single Select |
|  |  | Islamic | | |  |
|  |  | Primary | | |  |
|  |  | Secondary | | |  |
|  |  | Higher | | |  |
|  |  | Mother not present | | |  |
| Q22 | ASK: What is the occupation of the mother? | Not employed | | | Single Select |
|  |  | Housewife | | |  |
|  |  | Self-employed | | |  |
|  |  | Employed by other business | | |  |
|  |  | Professional e.g. Teacher/Lawyer | | |  |
|  |  | Casual Labourer | | |  |
|  |  | Farmer | | |  |
|  |  | Other  Mother not present | | |  |
| **[End of each household]** | | | | | |

**Supplementary Material 3. Red Cross Polio Post-campaign Survey Questionnaire**

| **No** | **Question** | **Responses** | | | **Response Type** |
| --- | --- | --- | --- | --- | --- |
| Q1 | Select Zone | [zones] | | | Single Select |
| Q2 | Select Woreda / County | [woredas / county] | | | Single Select |
| Q3 | Collect GPS position | [gps] | | | [gps] |
|  | | | | | |
| **INFO: Please obtain the following information from the village chief / head** | | | | | |
|  | | | | | |
|  |  | |  | |  |
| Q4 | ASK: What is the name of the settlement? | |  | | String |
| Q5 | ASK: Did the population group receive Polio vaccination during the last round? | | Yes / No | | Boolean |
| Q6 | ASK: What type of population group live here? | | Permanent (e.g. village) | | Single Select |
|  |  | | Semi-permanent (e.g. seasonal settlement) | |  |
|  |  | | Temporary (e.g. refugee, displaced) | |  |
| Q7 | ASK: What type of temporary population groups are present? | | Refugee | | Single Select |
|  |  |  | Internally Displaced Person | |  |
|  | ***[If response to Q6 is Temporary]*** | | Nomadic | |  |
| Q8 | ASK: How long has the population been living in the current location? | | Less than 1 month | | Single Select |
|  |  |  | 1 month to 6 months | |  |
|  |  | | 6 months to 1 year | |  |
|  |  | | More than one year | |  |
| Q9 | ASK: Is there a history of travel of this population group in the last month? | | Yes / No | | Boolean |
| Q10 | ASK: Where has the population travelled in the past month?  ***[If response to Q9 is Yes]*** | | Within the Woreda / County | | Single Select |
|  |  |  | Within the Zone | |  |
|  |  | | Within the Region  Across an international border | |  |
| Q11 | ASK: What are the ethnicities of people who live here? | | [ethnicities] | | Multiple Select |
| Q12 | ASK: How many households are present here? | | | | Integer |
| Q13 | ASK: What is the estimated population that live here? | | | | Integer |
|  |  |  | | |  |
| **INFO: We will now survey 10 households. Start from the centre of the population group and spin a bottle to determine your direction.** | | | | | |
| **INFO: Start with the first household closest to the centre of the population group.** | | | | | |
|  |  |  | |  | |
| **[For each household]** | | | | | |
| Q14 | CHECK: Does a child under five live in household [x]? | Yes / No | | | Boolean |
| Q15 | ASK: How many children under five live in household [x]? | | | | Integer |
| **[For each child in the household]** | | | | | |
| Q16 | ASK: What is the age group of child [x]? | Less than 6 months | | | Single Select |
|  |  | 6 months to 1 year | | |  |
|  |  | 1 to 2 years | | |  |
|  |  | 2 to 5 years | | |  |
| Q17 | ASK: Was child [x] vaccinated for Polio during the last round? | Yes / No | | | Boolean |
| Q18 | ASK: Why was child [x] not vaccinated during the last round? | Child / Parents Not Available | | | Single Select |
|  | ***[If response to Q17 is No]*** | Team did not Visit the house | | |  |
|  |  | Child available in house but not vaccinated | | |  |
|  |  | Child Asleep | | |  |
|  |  | New Born | | |  |
|  |  | Visitor | | |  |
|  |  | Child sick | | |  |
|  |  | Other | | |  |
|  |  | Refused | | |  |
| Q19 | ASK: Why was child [x] refused to be vaccinated for Polio during the last round? | Religious | | | Single Select |
|  |  | No faith in vaccine | | |  |
|  | ***[If response to Q18 is Refused]*** | Decision maker not at home | | |  |
|  |  | Fatigue | | |  |
|  |  | Rumour | | |  |
|  |  | Demand for other services | | |  |
|  |  | No response / Other | | |  |
| **[End of each child in the household]** | | | | | |
|  | | | | | |
| Q20 | OBSERVE: Was the house marked by vaccination team? | Yes / No | | | Boolean |
| Q21 | OBSERVE: What material is the floor of the house made of? | Mud | | | Single Select |
|  |  | Cement | | |  |
|  |  | Other | | |  |
| Q22 | OBSERVE: What material are the walls of the house made of? | Earth | | | Single Select |
|  |  | Stone | | |  |
|  |  | Iron Sheets | | |  |
|  |  | Cement | | |  |
|  |  | Timber/Wood | | |  |
|  |  | Bricks/Blocks | | |  |
|  |  | Cardboard/Cartons | | |  |
| Q23 | OBSERVE: What material is the roof of the house made of? | Iron Sheets | | | Single Select |
|  |  | Cement | | |  |
|  |  | Grass/Makuti | | |  |
|  |  | Tiles | | |  |
|  |  | Timber/Wood | | |  |
|  |  | Other | | |  |
| Q24 | ASK: Was the household aware of the last polio campaign before start of it? | Yes / No | | | Boolean |
| Q25 | ASK: What was the household source of information about the last polio campaign? | Radio | | | Single Select |
|  |  | TV | | |  |
|  | ***[If response to Q24 is Yes]*** | MoH Health Staff / Vaccinators | | |  |
|  |  | Women / Youth group activities | | |  |
|  |  | Red Cross volunteers | | |  |
|  |  | Other Community mobilisers | | |  |
|  |  | Megaphones | | |  |
|  |  | Mosque Announcements / Religious leaders | | |  |
|  |  | SMS / Mobile | | |  |
|  |  | Other | | |  |
| Q26 | ASK: What assets do the household have: | Electricity | | | Multiple Select |
|  |  | Radio | | |  |
|  |  | Television | | |  |
|  |  | Phone | | |  |
|  |  | Fridge | | |  |
|  |  | Solar Panel | | |  |
|  |  | None | | |  |
|  |  | No response | | |  |
| Q27 | ASK: What is the highest level of school the mother has completed? | No formal education | | | Single Select |
|  |  | Islamic | | |  |
|  |  | Primary | | |  |
|  |  | Secondary | | |  |
|  |  | Higher | | |  |
|  |  | Mother not present | | |  |
| Q28 | ASK: What is the occupation of the mother? | Not employed | | | Single Select |
|  |  | Housewife | | |  |
|  |  | Self-employed | | |  |
|  |  | Employed by other business | | |  |
|  |  | Professional e.g. Teacher/Lawyer | | |  |
|  |  | Casual Labourer | | |  |
|  |  | Farmer | | |  |
|  |  | Other | | |  |
|  |  | Mother not present | | |  |
| **[End of each household]** | | | | | |
